# The protective effect of longer duration of breastfeeding against pregnancy-associated triple negative breast cancer

**DOI:** 10.18632/oncotarget.9690

**Published:** 2016-05-29

**Authors:** Wael M. ElShmay

**Affiliations:** ^1^ Cancer Institute, University of Mississippi Medical Center, Jackson, MS, USA

**Keywords:** parity associated breast cancer, oncogene escape, triple negative breast cancer, metastasis

## Abstract

Parity associated breast cancer (PABC) often diagnosed within the 2-5 years after a full term pregnancy. PABC is usually present with more advanced, poorly differentiated, high-grade cancers that show shorter time to progression and often of the triple negative breast cancer (TNBC) subtype. Data from around the world show that pregnancy-associated TNBC is independently associated with poor survival, underscoring the impact of the pregnant breast microenvironment on the biology and consequently the prognosis of these tumors. Although it is not yet clear, a link between pregnancy-associated TNBCs and lack or shorter duration of breastfeeding (not pregnancy *per se*) has been proposed. Here, we present epidemiological and experimental evidence for the protective effect of longer duration of lactation against pregnancy-associated TNBCs, and propose a putative molecular mechanism for this protective effect and its effect in eliminating any potential TNBC precursors from the breast by the end of the natural breast involution.

## EPIDEMIOLOGICAL EVIDENCE FOR THE PROTECTIVE EFFECT OF LONGER DURATION OF BREASTFEEDING AGAINST PREGNANCY-ASSOCIATED TNBCS

Although pregnancy protects against the development of ER-positive breast cancers by reducing the level of circulating or local estrogen [1], epidemiological evidences suggest positive impact on the formation of TNBCs, especially in women lactated for shorter durations and mostly in African-American [2] and premenopausal Hispanic [3] women.

Until recently, longer duration of breastfeeding was a natural continuation of childbearing in the Middle East, Africa and Southeast Asia, where > 90% of women used to breastfeed their infants for > 12 months. This trend was positively correlated with significant protection against pregnancy-associated TNBCs in these regions. For example, two recent case-control studies, one conducted in Tunisia between 2006-2009 on 400 cases and 400 controls [4], and another conducted in South India between 2002-2005 on 1,866 cases and 1,873 controls [5], showed that longer lifetime duration of breastfeeding was inversely associated with breast cancer risk among premenopausal women [6-11].

A case-case population study conducted in Spain on 510 women diagnosed with operable breast cancers between 1997 and 2010 showed a lower proportion of TNBCs compared to luminal A cases among women who breastfed ≥ 7 months [12]. Additionally, in a meta-analysis of 47 epidemiological studies conducted in 30 countries that analyzed data on breastfeeding patterns for 50,302 women with invasive breast cancer and 96,973 controls, an inverse correlation between longer duration of breastfeeding and breast cancers in pre- and postmenopausal women, was reported [1]. In this analysis, the relative risk for breast cancer was shown to decrease by ~4.3% (95% CI 2.9-5.8; *p < 0.0001*) for every 12 months of breastfeeding, underscoring the protective effect of longer duration of breastfeeding against pregnancy-associated TNBCs [13-21].

## MAMMARY GLAND DEVELOPMENT DURING PREGNANCY, LACTATION, AND INVOLUTION

To understand the biology behind this protective effect, we must first understand the biology of the mammary gland development. The mammary epithelium is a highly heterogeneous and dynamic tissue containing several types of cells that differ significantly in their proliferative and differentiation capacities [22-25]. The mammary epithelium consists of an outer layer of basal, contractile myoepithelial cells, and an inner layer of ductal and alveolar, milk-secreting luminal cells [22].

**Figure 1:**
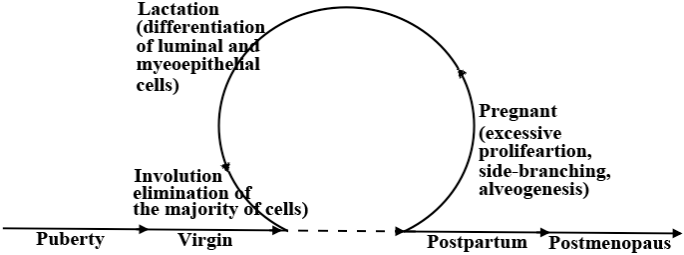
Stages of postnatal mammary gland development After the developmental changes occurring during puberty, the adult mammary gland can undergo several rounds of development during pregnancy, lactation, involution, postpartum and postmenopausal. Highlighted are the known key events occurring in each stage. Excessive proliferation and side-branching occur during pregnancy, and differentiation occurs during lactation and finally during involution almost all cells die by apoptosis and a mammary gland is re-organized into a pre-pregnancy state.

During pregnancy, extensive expansion and morphological changes prepare the mammary gland for lactation [26]. Side-branching and alveologenesis in response to progesterone (P) alone or in combination with prolactin (PRL) [27-29] generates secretory alveoli capable of producing milk [22] (Figure 1). Moreover, oxytocin released by suckling causes contraction of the surrounding myoepithelial cells that moves milk through the ductal tree to the nipple [22]. Additionally, during lactation significant changes to the vasculature, the adipose tissue and the extracellular matrix within the mammary gland trigger terminal differentiation of the majority of mammary cells [30-32] (Figure 1). Finally, during involution (the post-lactation weaning) all terminally differentiated mammary cells are eliminated by cell death and the mammary gland undergoes a significance re-organization that returns it to pre-pregnancy quiescent state [33-35] (Figure 1).

**Figure 2:**
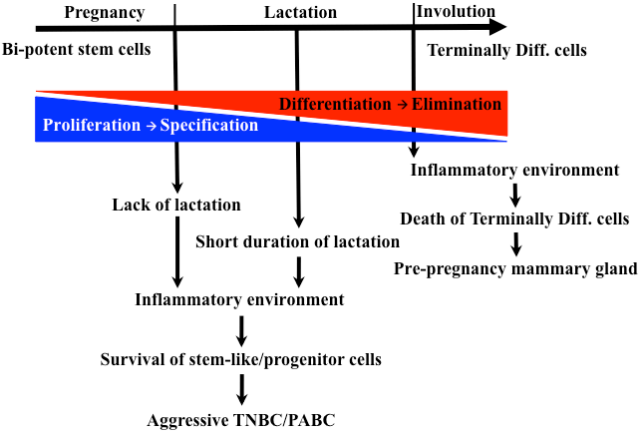
Evolution of mammary gland and pregnancy-associated TNBCs The different stages of parity (pregnancy, lactation and involution) super-imposed on the different events occurring. The proliferation and specification of the mammary gland occurs during pregnancy and seizes during lactation. In contrast, differentiation of these cells during lactation leads to their elimination by apoptosis during involution. It is proposed that the inflammatory environment associated with involution that induced normally after longer lactation periods would target terminally differentiated cells and kill them. In contrast, forced involution induced by lack or short duration of breastfeeding could lead to inflammatory environment that could promote the survival and expansion of less differentiated cells and formation of aggressive TNBCs/PACSs.

On the cellular level, during pregnancy specification steps induce bi-potent stem cells located within the basal cell layer to give rise to uni-potent luminal or myoepithelial progenitor cells [22-25, 36]. Thereafter, intrinsic (e.g., specific transcriptional factors) and extrinsic (e.g., interactions with the local microenvironment) promote differentiation in these cells and formation of fully functional gland (Figure 2). For instance, GATA3 [37], STAT5a [38], Myc [39] and LRG5 [40] regulate differentiation of luminal progenitors into luminal epithelial cells, whereas p63 [41, 42], serum response factor (SRF) [43] and SLUG [44] regulate differentiation of basal progenitors into myoepithelial cells. Additionally, interactions with the extracellular matrix [ECM] proteins in general and the basement membrane [BM] proteins in particular [45, 46], and the infiltrated immune cells are also major drivers of progenitor differentiation. In fact, correct tissue architecture, including ECM organization and stiffness together with the reservoir of growth factors, cytokines and proteinases within it are essential mediators of mammary gland development and proper function [34].

## MOLECULAR BASIS FOR MAMMARY GLAND DEVELOPMENT DURING PREGNANCY, LACTATION AND INVOLUTION

**Figure 3:**
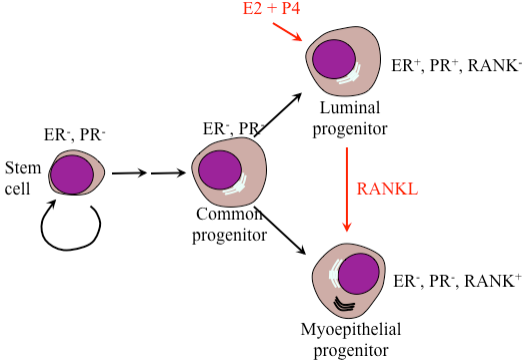
Model of the differentiation hierarchy within mammary epithelium Stem cells negative for ER and PR reside within the basal layer of the mammary gland. Upon initiation of pregnancy, these cells undergo several rounds of proliferation and specification to produce common bipotent progenitor cells that further specified to become luminal specific and basal specific progenitor cells. Luminal progenitors proliferate in response to P4 and E2. However, the ER-, PR-negative but RANK-positive meyoepithelial progenitor cells expand in response to RANKL expressed by luminal progenitor cells.

Normal mammary gland contains PR (and ER)-positive and PR (and ER)-negative (stem-like/progenitor) cells normally border each other with adherence junctions [47] (Figure 3). *In vitro*, PRL/PRLR signaling through MAP-kinase regulates PR expression [48]. Deletion of the transcription factor CCAAT/enhancer binding protein c/EBPβ results in a severe inhibition of lobulo-alveolar development in mouse mammary gland. While the number of PR-positive cells was elevated in the mammary glands of c/EBPβ^-/-^ mice, no change in c/EBPβ mRNA level was observed in the mammary glands of PR^-/-^ mice, suggesting that PR acts in parallel to or downstream of c/EBPβ [49]. During early development, the distribution of PR-positive cells shifts from a uniform to a non-uniform pattern in wild-type nulliparous mice and not nulliparous c/EBPβ^-/-^ mice [49]. PR-positive cells were non-proliferative in wild-type mice but proliferative in c/EBPβ^-/-^ mice [50]. These data suggest that c/EBPβ controls mammary gland cell fate through controlling expression of molecular markers, such as PR, that induce proliferation in alveolar progenitor cells *via* juxtacrine mechanisms.

During pregnancy, luminal PR^+^/PRLR^+^ cells produce and secrete receptor activator of nuclear factor κB ligand (RANKL) that drives expansion of the RANK (a TNF-related molecule)-expressing PR^-^/PRLR^-^ progenitor cells through juxtacrine signaling [50] and thus mammary glands lobuloalveolar structure formation (Figure 3). RANKL/RANK stimulation induces Inhibitor of DNA binding (Id) 2 phosphorylation at serine 5, and nuclear retention [51-53]. In the nucleus, the helix-loop-helix (HLH) Id proteins that lack the basic domain important for DNA binding interaction with members of the basic helix-loop-helix (bHLH) transcription factors negatively regulates cell lineage commitment and differentiation, while positively regulates cell proliferation in mammary epithelial progenitor cells [51], indicating a principle role for Id2 in pregnant mammary glands.

Data from human breast cancer cell lines, patient tumor samples and clinical studies indicate that P/PR and PRL/PRLR signaling pathways contribute to early stage human breast cancer progression. On the other hand, loss of PR/PRLR expression in primary tumors is associated with a less differentiated more invasive phenotype and worse prognosis, suggesting a tumor suppressive role for PR/PRLR during later stages of tumor progression. Additionally, over-activation of RANKL/RANK signaling and Id2 overexpression enhances mammary tumor formation by increasing the proportion of basal/bi-potent cells and sustained inhibition of differentiation of these cells towards milk producing mammary cells as well as predicts poor prognosis in breast cancer [50-53].

## MOLECULAR BASIS FOR LONGER DURATION OF BREASTFEEDING PROTECTION AGAINST PREGNANCY-ASSOCIATED TNBCS

Involution whether induced after a prolonged (normal involution) or short (forced involution) breastfeeding is complex process encompasses in addition to extensive death of milk-producing epithelial cells, the removal of these dead cells, residual milk and debris, controlled by an influx of phagocytic cells, such as macrophages into the involuting mammary gland. Indeed, compared to nulliparous glands, gene expression signature from parous glands is enriched in inflammatory and immune response genes [54-57]. Interestingly, in early involution, viable epithelial cells are utilize­d as phagocytes until professional macrophages enter the involuting mammary gland [56].

During involution, activation of signaling pathways, such as STAT3 and NF-κB triggers important inflammatory signaling pathways [60] that generate pro- as well as anti-inflammatory genes in involuting mammary gland, probably to ensure that overt inflammation does not occur [61]. Additionally, cell engulfment induces production of anti-inflammatory cytokines such as TGF-β [56], essential for ensuring that involution proceeds without inflammation. Involution also entails breakdown of extracel­lular matrix, remodeling of blood vessels and re-differentiation of adipocytes to regenerate the fad pad [58, 59].

This pro-tumorigenic inflammatory microenvironment most definitely affects differentiated cells and progenitor cells differently. Indeed, evidences do exist implicating the inflammatory environment in the death of differentiated cells, but survival, expansion and enhanced aggressiveness in progenitor cells [36]. Implantation of breast cancer cells into mammary glands of mice undergoing involution accelerated tumor formation and metastasis [62-64]. TNBCs are particularly prone to inflammatory environment, and thus the presence of basal progenitor cells in an involution-mimicking environment initiated by lack or shorter lactation period enriched with pro-inflammatory cytokines could exacerbate TNBCs [66-69]. Lack or delayed differentiation of progenitor mammary epithelial cells imposed by the lack or the shorter periods of breastfeeding could account for post-pregnancy TNBCs [66-70].

Thus, the excessive proliferative expansion of progenitor cells occurring within breast lobules during pregnancy ought to be followed by longer periods of breastfeeding to allow a progressive loss of these progenitor cells status to terminal differentiation. Lack of such differentiation could lead to retention of progenitor basal cells within the breast lobules, which after exacerbation by the inflammatory environment induced during forced weaning could convert them into precursors for post-pregnancy TNBCs [71-78].

## THE “ONCOGENE ELIMINATION HYPOTHESIS”

**Figure 4:**
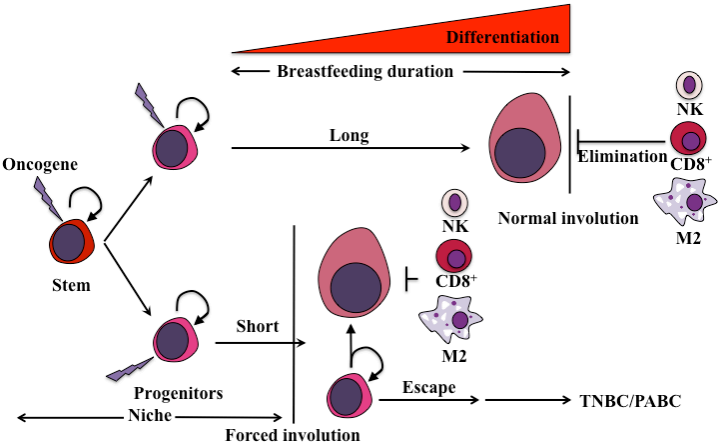
Proposed model for the generation of pregnancy-associated TNBCs We propose that an oncogene(s) is overexpressed (lighting bolt) by pregnant and lactating cells to perform a specific function such as excess proliferation and survival. Since the cells overexpressing that oncogene must die before the mammary gland returns to pre-pregnancy stage, two outcomes depending on the duration of breastfeeding could be envision. The first is an oncogene-elimination outcome, in which during the longer duration of breastfeeding ( > 12 months) all oncogene-overexpressing cells undergo a full differentiation program. These terminally differentiated mammary cells are also immune-presenting and therefore will be detected and eliminated by the immune cells infiltrating the mammary gland during normal involution and replaced by newly low oncogene-expressing cells. The second is oncogene-escape outcome, in which during shorter (or no) duration of breastfeeding ( < 12 months), many of the oncogene-overexpressing cells are still in the stem/progenitor stage. During forced involution and the influx of immune cells that ensues, oncogene-expressing terminally differentiated cells will be recognized and eliminated as described above, whereas the oncogene-expressing stem/progenitor cells will escape immune system recognition and elimination and instead thrive, flourish and become pregnancy-associated TNBC precursors in this inflammatory microenvironment, that develop 2-5 years following this pregnancy.

All known oncogenes have normal functions when expressed in cells at normal levels. Some oncogenes could also be overexpressed during a specific stage of development to perform a specific function such as enhanced proliferation and survival of target cells. Many proliferation-inducing oncogenes block differentiation. Conversely, differentiation signals that conflict with the effect of these oncogenes promote apoptosis in these oncogene-expressing cells [39].

we propose that upon initiation of pregnancy, a specific oncogene(s) expression is increased in cells to promote their proliferation and/or survival. Cells expressing this oncogene must be eliminated at the end of lactation during involution. However, longer duration of breastfeeding that steers all mammary cells to terminal differentiation could specifically kill these oncogene-expressing cells by the involution environment and thus rid the mammary gland from these pro-tumorigenic cells (Figure 2). Lack or shorter periods of breastfeeding could lead to persistence of some oncogene-overexpressing cells with tumorigenic potential, especially in the inflammatory microenvironment during early weaning leading to formation of pregnancy-associated TNBCs [9-11] (Figure 2). Thus, the molecular potency of a specific oncogenic insult, combined with the stage in cellular differentiation and the effect of the pro-tumorigenic microenvironment during forced involution could have a profound effect on the post-pregnancy TNBCs that ensue [79-81]. We call this the “oncogene elimination hypothesis”, in which two mutually exclusive outcomes are proposed:

1. The elimination outcome mostly present in the mammary glands of women who lactate for longer periods of time. In these glands, fully/terminally differentiated cells overexpressing this oncogene are also cells that express immune cells recruiting factors. These cells will also be tumor-specific peptides presenting (*via* MHC class Ia molecules) cells. Upon normal involution, these cells will be easily detected and eliminated by the infiltrated immune cells leading to restoration of normal tissue containing no oncogene overexpressing cells and low chance of post-pregnancy TNBCs formation [82] (Figure 4).

2. The escape outcome mostly present in the mammary gland of woman that do not breastfeed or breastfeed for shorter duration of time. In these glands, the accumulation of mix of fully differentiated cells and cells with stem-like/progenitor properties occurs. Upon forced involution an influx of immune cells into the mammary gland will still occur. As described above, fully/terminally differentiated cells will be recognized and eliminated. On the other hand, stem-like/progenitor cells overexpressing the oncogene also express a multitude of immunosuppressive factors that could protect them in this environment. These cells that escaped the immune clearing and overexpress the oncogene will ultimately expand generating less-immunogenic or more immunosuppressive subclones that eventually acquire the ability to expand independently of their niches leading to the outgrowth of poorly differentiated pregnancy-associated TNBCs [82] (Figure 4).

## PUBLIC-HEALTH IMPLICATIONS

Association analyses between reproductive factors and risk or odds of developing ER^+^/PR^+^, HER2^+^, and TNBC tumors showed strong association between ER^+^/PR^+^ cancers and nulliparity, current use of menopausal hormone therapy, increased age at first child birth and decreased age at menarche. In contrast, lactation was inversely associated with ER^+^/PR^+^ tumors but positively associated with TNBCs [83-86]. No remarkable associations for HER2^+^ breast cancers were evident. All the studies suggest that major reason for the high incidence rates of breast cancer in the developed countries is the short duration of breastfeeding. If women in these countries had longer lifetime durations of breastfeeding typical of women in the developing countries, the cumulative incidence of breast cancer will be reduced by more than half by age 70 years. These analyses showed a 50% reduction in the odds of TNBC for younger women ( < 45) who had breastfed greater than 12 months compared to those who had never breastfed.

To expect that the trend will change anytime soon is unrealistic. More productive is to identify the mechanism of the protective effect of breastfeeding and prevent breast cancer by mimicking its effect therapeutically. In the meantime, important reductions in breast-cancer incidence could be achieved if women considered breastfeeding each child for longer than 12 months [87, 88]. There are obvious economic and social consequences to prolonging breastfeeding; there are also benefits to the mother, as well as the known benefits to the child [89, 90].
